# Are there similarities between the Corona and the climate crisis?

**DOI:** 10.1007/s13412-021-00666-5

**Published:** 2021-01-29

**Authors:** Robert C. Schmidt

**Affiliations:** FernUniversität, Hagen, Germany

**Keywords:** Climate change, Corona crisis, Democracy, Incentives, Constitution

## Abstract

In this short paper, I look back at the early stages of the Corona crisis, around early February 2020, and compare the situation with the climate crisis. Although these two problems unfold on a completely different timescale (weeks in the case of Corona, decades in the case of climate change), I find some rather striking similarities between these two problems, related with issues such as uncertainty, free-rider incentives, and disincentives of politicians to adequately address the respective issue with early, farsighted and possibly harsh policy measures. I then argue that for complex problems with certain characteristics, it may be necessary to establish novel political decision procedures that sidestep the normal, day-to-day political proceedings. These would be procedures that actively involve experts, and lower the involvement of political parties as far as possible to minimize the decision-makers’ disincentives.

Both anthropogenic climate change and the Corona crisis are problems characterized by a delay between the societal efforts to tackle the respective problem, and the benefits from these efforts. In each of these cases, global harmonized efforts, and cooperation are needed, as well as unilateral action. As an economist, I have analyzed the climate change problem for years, focusing among other things on the incentives of governments to adopt suitable policies to tackle the issue, paying attention also to various obstacles that may hamper effective climate policy. Regarding the nature of these two problems, I find some rather striking similarities. It is conceivable that the Corona crisis may provide us with valuable insights about the way in which societies (and in particular democracies) deal with problems of a certain kind. If that holds true, then it may be possible to draw some conclusions on the way in which democratic decision processes might be structured in order to become more effective. In other words: the Corona crisis may guide us towards systemic improvements in democracies that may help us to tackle other problems, such as climate change. I personally hope, that after the Corona crisis, our societies will not simply return to their business-as-usual, where political decision-making often seems to focus primarily on (short-term) economic success. Changes in the political system itself will be needed, in order to overcome a lack of incentives for the politicians to address some severe, often long-term challenges that democracies have so far failed to address adequately.

An obvious problem that makes it difficult for politicians to deal with climate change is the time lag between any efforts to lower the emissions of greenhouse gases, and the benefits resulting from those efforts, in terms of reduced climate damages. The latter are mostly enjoyed by future generations. Also with Corona, there is a delay between the efforts to contain the spread of the virus, and the societal benefits from these efforts. However, while the timescale of climate change is decades, the spread of the Corona virus, which may be dubbed a “mini climate change”, virtually moves in quick motion: the timescale is measured in weeks rather than decades. Nevertheless, even here, the time lag presents a huge problem for effective political decision-making. In particular, harsh policy measures for a limited period of time, beginning in February 2020, might have permitted to prevent the spread of the new Corona virus (Sars-CoV-2/Covid-19) in countries that were not yet affected by the epidemic, and possibly even to eradicate the disease globally, as accomplished with Sars and Mers several years ago.

To this end, it would have been necessary to reduce the mobility of millions of people, promptly after the outbreak of the epidemic in China. The goal of such measures would have been to prevent the virus from entering the country in the first place. To this end, it might have been worth considering cancellations of flights or trains, and sending all persons entering from the affected areas or countries into a sufficiently long mandatory quarantine, testing them for the virus before leaving the quarantine.^1^ These measures might have allowed to prevent the spread of the virus into new countries.^2^ However, at the time (in early February 2020), these measures might have been regarded as draconian and exaggerated and would probably have been very unpopular. Yet, it is conceivable, that with such measures, the spread of the virus might have been contained, or the virus even been eradicated in the end, if a sufficient number of countries would have adopted similar measures and cooperated in dealing with this issue. The damages (by deaths and economic distortions) that the world probably has to face now may be orders of magnitude higher than those arising from such a temporary (even if massive) restriction of human freedom of movement for a limited period of time at an earlier date. This would have required a high degree of forward-looking political decision-making, at a time when the majority of the people in countries not (yet) affected by the virus presumably would not have understood the rationale behind such harsh measures. In analogy with the climate crisis, also with Corona, such early efforts might have helped to prevent future damages drastically.^3^

In March 2015, Bill Gates (founder of the Microsoft company) held a talk in Vancouver, BC, Canada on the possible risks of future pandemics.^4^ In the light of the current Corona crisis, his talk seems almost prophetic, as it anticipated many of the problems that the world is currently struggling with. Among his policy recommendations is that health systems should be strengthened, that a medical reserve corps should be established, and that R&D should be fostered in the area of vaccines and diagnostics. In my opinion, one should add to his (otherwise very reasonable) list of measures an early action plan, to rapidly shut down travel to and from countries already affected, in order to use the possibility to prevent an epidemic that is developing elsewhere from arriving in countries not yet affected (if that is possible), or to minimize the number of cases so that the spread of the virus may still be contained with measures that are less drastic than the lock-downs that are currently pursued by many countries around the globe.

The next analogy between Corona and the climate crisis is that in both cases, international cooperation is needed. Just like no single country can stop climate change, no single country can prevent the spread of (or even eradicate) the Corona virus. In the context of the climate problem, we (economists) often use the term “carbon leakage” (see Branger and Quirion 2014). This means that unilateral efforts of an individual country to reduce the emissions of greenhouse gases are undermined, if other countries do not move along, as firms may relocate their production, and domestic firms may lose their international competitiveness. Thus, emissions are effectively shifted from one country to another instead of being reduced globally under unilateral emission mitigation policies, unless additional instruments are used that specifically address leakage, such as the implementation of a border carbon adjustment scheme. Otherwise, carbon leakage reduces the incentives of any single country to invest in climate change mitigation in the first place. And it reinforces the so-called “free-rider incentive” that characterizes the climate problem from a game-theoretic point of view: instead of becoming a proactive country, each country rather has incentives to remain inactive and let other countries go ahead, by investing little to reduce the emissions domestically.^5^ This is because any unilateral efforts to reduce greenhouse gas emissions will in the end benefit all countries in the world, while the costs accrue mostly to the domestic economy and society. Also for the Corona problem, there is some kind of a “leakage” problem, as the virus may spread across borders. However, while the leakage of emissions in the climate context intensifies if a single country steps up its carbon mitigation efforts, the opposite holds true for Corona, where a proactive country may help to reduce the spread of the virus across borders. Furthermore, one may also identify some sort of a free-rider incentive with respect to Corona: if a large number of other countries step up their efforts to prevent the spread of the virus, for any other country, it should be easier to contain the spread of the virus domestically, because it becomes less likely that the virus enters the country from elsewhere. Recall, that my considerations here refer to the situation around early February 2020, and not to the situation several weeks later, when the epidemic had already turned into a pandemic.

Another obstacle to an effective climate protection policy is uncertainty. This concerns both the extent of the future damages to be expected^6^, as well as the costs of climate change mitigation. Nobody can predict with certainty how costly it would be to reach, say, the 1.5 °C target, and with which technologies exactly this may be achieved. This is similar with Corona: on the one hand, the damages of a pandemic are hard to predict. These are ranging from thousands, possibly hundreds of thousands of deaths around the globe (conceivable ranges when looking at the situation from the perspective of early 2020), all the way up to a possible global economic crisis. On the other hand, it would also have been very hard to predict the costs of harsh, immediate measures to prevent the spread of the virus to countries not (yet) affected by it early on, beginning in February 2020, with the goal to prevent the pandemic, and possibly to eradicate the disease. And last but not least, it would have been uncertain if the virus could have been contained if such measures had been taken. This might have been achieved, but only with some (unknown) probability. There is again an analogy with the climate change problem: due to tipping points in the climate system, climate change may get out of control. This can happen, but this is not for sure. This also holds for the Corona problem: the epidemic can get out of control (looking at it from the perspective of early February 2020), but this was not for sure. In both cases, the different kinds of uncertainties surrounding the problem make it much harder for politicians to implement strict policy measures early on (even if these would be useful).

Yet another dimension of the climate change problem, where some similarities with the Corona crisis may be found, is the public perception and media coverage of the respective problem, as well as the impact of politicians, political parties, or interest groups (e.g., lobbyists) who are trying to influence the media coverage in line with their own interests or (potentially biased) beliefs.^7^ A well-documented fact is that at least some political parties take an active role in conveying their disbelief in climate science, and voters extract information from parties’ publicly stated opinions or policy platforms (e.g., Hornsey et al., 2016; Guber, 2013).^8^ A striking fact is also, that—as early as in 1982—scientists working for Exxon provided a forecast of the expected increase of the atmospheric CO_2_ concentration and the resulting global warming that has proven to be remarkably accurate until today.^9^ By contrast, in public communications, the company consistently casted doubts on whether climate change is “real, human-caused, serious, and solvable” (Supran and Oreskes 2017). Remarkably, one of the key demands of the Fridays-for-Future movement is to “unite behind the science”. Regarding the Corona crisis, the harmful effects that negligence of scientific advice^10^ by politicians can have for societies is illustrated starkly by the experiences of the USA with the crisis. “Donald Trump was warned at the end of January by one of his top White House advisers that coronavirus had the potential to kill hundreds of thousands of Americans and derail the US economy, unless tough action was taken immediately, new memos have revealed…. They show that even within the Trump administration alarm bells were ringing by late January, at a time when the president was consistently downplaying the threat of Covid-19.” (Source: The Guardian).^11^^,^^12^ However, because these two problems unfold on completely different time scales, some of the errors of policy-makers in handling the Corona crisis become visible after several weeks, whereas the dramatic lack of adequate policy action for tackling climate change today and over the past decades may become evident only decades from now. The effects may then be much more devastating than the damages from the Corona crisis, as irreversible tipping points are involved that have the potential to trigger catastrophic climate change that can no longer be controlled.^13^

This brings me to the final analogy between these two problems that I want to point out: a systemic lack of incentives for politicians to adequately address the respective problem. Let us first take a look at the climate change problem. The problem of anthropogenic climate change has been sufficiently well documented scientifically since 3 or 4 decades ago, in order to justify the implementation of tough policies with the goal to lower the greenhouse gas emissions and, hence, damages from climate change in the future. As early as in the beginning of the 1990s, strict climate change mitigation policies would have been very well justified, and indeed necessary from the scientific standpoint. However, global emissions have been rising until today, and it must be admitted that also democracies have by and large failed to address the climate change problem adequately so far.^14^ Climate change is a problem, that by its very nature would have required early policy action (and still does), at a time when no (severe) climate damages were visible yet. Politics, and this holds true also for democracies, often seems to address problems only when the damages already start to become obvious to (almost) everybody, hence, when the issue becomes pressing, as is the case with climate change today. However, from a scientific standpoint, this is by far too late (several decades too late). In this sense, we can speak here about a systemic failure of democracies (and other political systems), that have failed to address the problem adequately. Hence, we must also look at the functioning of political systems themselves, in order to gain a deeper understanding of the systematic failure of democracies to adequately address the climate change problem—“systematic” because it could be observed over several decades and in many countries around the globe.^15^ The underlying problem appears to be, that the politicians are lacking the right incentives to implement early, farsighted policies. Such policies are initially unpopular and, hence, politicians who implement them have to fear for their political careers, since the broad population cannot fully comprehend the necessity of the measures when the damages are not yet visible. This would require a sufficient knowledge of the scientific evidence and literature that generally only experts have. As a result, the incentives for politicians are such that they tend to ignore the problem at the early stage, even if proactive policy measures would already be necessary at this point.

This also applies for Corona although this problem is unfolding in fast motion (compared to climate change). Also here, there was a lack of incentives for politicians to tackle the problem at the (very) early stage. With an early massive intervention to stop the global epidemic and possibly eradicate the virus, by restricting travel to and from countries that were at the time affected by the epidemic, and by putting any remaining persons who recently visited those areas into a mandatory quarantine when entering the country, the politicians in charge would have been bound to lose: (1) had such measures been taken and been successful, many people would have considered the measures to be disproportional: “It wasn’t that bad after all – the policymakers were overreacting!”^16^ And (2) had they taken such tough and early measures without being able to stop the virus, the political damage might have been even bigger. Many people would have said that the policymakers had reacted out of proportion while the virus could not be stopped anyway. Since most countries failed to use the (very) early phase to contain Corona in order to avoid a pandemic, the policymakers can now even take the role of crisis managers.^17^ A cynic might say that the crisis first had to develop and become visible for everyone before political acting in a democracy could gain a majority. By contrast, early, farsighted policy-making, that might have been much more effective in preventing economic and health damages for the society in the longer run, was politically infeasible. As is the case with climate change, also for this problem, it is thus possible to identify systemic errors in democracies that seem to have their roots in a lack of incentives for the politicians to tackle certain issues based on scientific evidence, when the need for early action is not yet visible to a majority of the people.

In my opinion, problems such as climate change, insect die-off, plastic pollution, antibiotic overuse, and also epidemics, should not even be a part of the ordinary, everyday type of political decision-making. Instead, such problems should be treated fundamentally different from other problems, where the main conflict is often about a social balance between different parts of the society. One possibility to deal with those problems might be to establish an expert committee drawn from scientific institutions (like universities) that develops the key points of a catalogue of measures, or some minimum standards for policies, built upon scientific evidence and necessity. This would be more than just an advisory board. I rather suggest to bypass the normal political proceeding. Obviously, scientists alone cannot decide on laws. The democratic legitimation needs to be assured. However, this may be achieved in different ways, for example with the help of a citizens’ assembly (as was used, e.g., in Ireland), possibly combined with a referendum, or via the parliament.^18^ In the latter case, in my opinion a secret parliamentary ballot on the list of measures suggested by the scientists would be necessary, without any direct influence of the political parties to eliminate political disincentives as far as possible.

Moreover, I suggest the implementation of a regulated process to identify such problems that cannot be adequately dealt with in the ordinary run of parliamentary procedures, e.g., due to problems such as those outlined above (uncertainty, time-lags, incentive problems for politicians, etc.), and to separate them from the political day-to-day business. Especially in the context of climate protection, in my opinion, the policies should not even be dependent on which party (or parties) holds the power. Some problems, by their very nature, should be treated separately from the normal day-to-day political proceeding, because of their relevance for the entire society and based on scientific evidence. Thus, from my point of view, one might also think of changes or amendments to the constitution, in order to embed different procedures for dealing with such complex problems in the political system.

As disastrous as the present situation might be, it could also help to take a break, and encourage profound changes in the political system itself. Thus, we could be better prepared for future crises or be enabled to act early to avoid such crises, even if the broader population might not understand the necessity of the preventive measures at the early stage.

## Footnotes

1 “Just 273 of the 18.1m people who arrived in UK by air in run-up to lockdown were quarantined”. Source: The Guardian, www.theguardian.com/world/2020/may/05/just-273-people-arriving-in-uk-in-run-up-to-lockdown-quarantined (visited on July 14, 2020)

2 New Zealand’s experiences with the Corona crisis so far seem to point at the effectiveness of such measures. “New Zealand’s border is closed to most travellers and entry is strictly controlled. All arrivals are tested for COVID-19 and a 14-day managed quarantine or isolation is mandatory.” Source: New Zealand Immigration, www.immigration.govt.nz/about-us/covid-19/coronavirus-update-inz-response (visited on July 14, 2020)

3 Compound climate risks in the COVID-19 pandemic are discussed in Phillips et al. (2020).

4 TED talks, TED.com; www.youtube.com/watch?v=6Af6b_wyiwI (visited on March 24, 2020).

5 See Barrett (1994).

6 “The range of cross-sectional climate damage estimates is 0.1 to − 16.3% of GDP for temperature increases of 0.7–3.2 °C.” (Howard and Sterner 2017).

7 I am grateful to an anonymous reviewer for pointing this out.

8 See also Campbell and Kay (2014), Rafaty (2018), Shapiro (2016). The influence of social media in the context of misinformation about climate change is discussed also here: www.carbonbrief.org/guest-post-how-climate-change-misinformation-spreads-online (visited July 15, 2020).

9 It “includes a graph (see https://perma.cc/PH4X-ZJBA ) showing 'an estimate of the average global temperature increase' under the 'Exxon 21st Century Study-High Growth scenario'.” Source: Supran and Oreskes (2017).

10 See Hopf et al. (2019) on fake science and the “fake news/fake science cycle”.

11 www.theguardian.com/world/2020/apr/07/donald-trump-coronavirus-memos-warning-peter-navarro (visited on July 14, 2020)

12 Later, it seemed that there were efforts by the White House to discredit Dr. Anthony Fauci, director of the NIAID (National Institute of Allergy and Infectious Diseases). See www.theguardian.com/world/2020/jul/13/trump-administration-fauci-coronavirus-crisis , or https://twitter.com/hashtag/faucithefraud (both visited on July 15, 2020). On a more general note, populist leaders in various countries have been criticized for poorly handling the Corona crisis, see https://thewire.in/politics/trump-bolsonaro-jognson-modi-populist-leaders-covid (visited July 15, 2020). Misinformation in social media in the context of the COVID-19 pandemic is analyzed in Laato et al. (2020).

13 These (potential) tipping points comprise, among others, a possible die-off of the Amazonian rain forest triggered by global warming or by excessive deforestation, the thawing of permafrost and associated release of the greenhouse gas methane stored in it, melting of glaciers and ice shields, resulting in higher absorption of sunlight and hence, additional warming. These effects may be self-reinforcing and hence, have the potential to lead to a cascade of tipping points and an irreversible regime shift in the overall climate system (Barnosky et al. 2012, Lenton et al. 2019). The recent fires in Siberia, in the Amazonian rain forest, and in Australia give a first impression that such irreversible processes may already have started, particularly with the “Arctic 'warming faster than anywhere' on Earth”, see www.dw.com/en/siberia-fires-permafrost/a-54120019 (visited July 14, 2020).

14 The widespread negligence of scientific evidence and necessity in climate policy is two-fold. On the one hand, the implemented policies are generally too soft, and fall short of reaching the mitigation targets that climate scientists propose (see, e.g., https://climateactiontracker.org/countries/eu/ , visited June 14, 2020). On the other hand, politicians usually do not implement the policy mix that a majority of environmental economists would suggest. In particular, it is widely acknowledged among experts on climate policy that any effective climate policy mix should comprise a uniform carbon price for all CO_2_ emissions of the economy. This can be implemented at low bureaucratic cost upstream, at the extraction and import of fossil fuels to reach full coverage of all CO_2_ emissions in the economy, and be combined with a border carbon adjustment scheme (BCA) to address competitiveness concerns that may be significant especially at carbon price levels that are sufficiently high to have a big dent in the emissions, see Schmidt (2019); English version: https://www.fernuni-hagen.de/mikrooekonomie/team/robert.schmidt.shtml (“A roadmap for the implementation of a carbon tax”).

15 I am convinced (but cannot prove), that the free-rider problem alone (Barrett 1994) cannot explain the systematic lack of effective climate policies around the globe, at least not to the extent that we observe. There are various aspects that offer ways out of short-sighted free-riding, ranging from (at least partial) altruism over a sense of moral obligation all the way to fairness. It seems obvious that every country should strive to decarbonize its economy in order to tackle climate change. A systemic lack of incentives for politicians to address the problem (to me) appears to be a plausible explanation for (at least part of) the political inaction.

16 There are indeed cases where scientists’ warnings were heeded and catastrophe averted, and then scientific concerns were portrayed as a “storm in a teacup” (I am grateful to an anonymous referee for pointing this out). Examples include the Millenium bug (Y2K-Bug), where major problems were predicted, but when the first of January 2000 arrived, “millennium bug has failed to deliver the disaster scenarios predicted by the gloomiest commentators” (http://news.bbc.co.uk/2/hi/science/nature/586620.stm , visited July 15, 2020), as well as CFCs (Chlorofluorocarbons), that were phased out via the Montreal Protocol due to their contribution to the depletion of the ozone layer. The Montreal Protocol is often seen as a major success in the area of international environmental cooperation, but critics argue that, among other things, the abundance of low-cost substitutes made this an easy-to-solve problem, in contrast to climate change (e.g., Barrett 2003, Victor 2011).

17 E.g., the popularity of the German government has increased during the Corona crisis, in spite of the fact that like many other countries, little action was taken during the early phase of the epidemic to prevent the virus from entering the country (such as mandatory quarantine measures in February 2020, for example). “Shaking off years of record-low popularity, Merkel’s center-right CDU/CSU bloc is now enjoying approval ratings of around 32 to 35%, some six to seven points higher than just a few weeks ago.” Source: AFP/The Local, 29 March 2020, www.thelocal.de/20200329/merkel-is-back-coronavirus-crisis-boosts-german-chancellor .

18 Information about the effectiveness of policy measures can raise their acceptability. For example, Carattini et al. (2017) find that “Providing information on the expected environmental effectiveness of carbon taxes reduces the demand for environmental earmarking.” Citizens’ assemblies may be one (out of many) possible ways to involve experts and other well informed people from outside of political parties in political processes. (Political parties may be prone to ideology and usually represent only a subset of societal interests—those of their respective constituencies). In the climate policy context, some forms of citizens’ assemblies are also used in the U.K. and in France, see e.g. www.theguardian.com/world/2020/jan/10/citizens-panels-ready-help-macron-french-climate-policies (visited June 14, 2020).
